# Supplementary material for: PTEN modulates urinary tract infection susceptibility and shapes urothelial antibacterial defenses
Source: Life Sci Alliance. 2025 Jul 23;8(10):e202503292. doi: 10.26508/lsa.202503292 (PMC12287727; doi:10.26508/lsa.202503292)
Supplement: Supplementary file 1 [file LSA-2025-03292_TableS1.docx]

| **Gene** | **Fold Regulation** | ***P*-value** | **Gene** | **Fold Regulation** | ***P*-value** | **Gene** | **Fold Regulation** | ***P*-value** |
| --- | --- | --- | --- | --- | --- | --- | --- | --- |
| *AKT1* | 1.17 | **0.034295** | *CXCL8* | -1.13 | **0.047951** | *NOD1* | -1.07 | 0.224357 |
| *APCS* | -1.44 | 0.456548 | *IRAK1* | 1.11 | 0.365187 | *NOD2* | 1.15 | 0.393691 |
| *BIRC3* | -1.09 | 0.163982 | *IRAK3* | 1.02 | 0.897103 | *PIK3CA* | -1.12 | **0.005702** |
| *BPI* | 1.78 | 0.165484 | *IRF5* | -1.1 | 0.117819 | *PRTN3* | 1.45 | 0.193803 |
| *CAMP* | 1.11 | 0.703467 | *IRF7* | -1.15 | 0.248298 | *PSTPIP1* | -1.01 | 0.992191 |
| *CARD6* | 1.18 | 0.069608 | *JUN* | -1.07 | 0.512443 | *PYCARD* | 1.22 | **0.036972** |
| *CARD9* | -1.30 | 0.74436 | *LBP* | 1.31 | 0.093185 | *RAC1* | 1.06 | 0.423183 |
| *CASP1* | 1.03 | 0.596029 | *LCN2* | -1.29 | 0.279979 | *RELA* | 1.14 | **0.041131** |
| *CASP8* | -1.01 | 0.857106 | *LTF* | 1.21 | 0.276712 | *RIPK1* | 1.02 | 0.782269 |
| *CCL3* | 1.19 | 0.596854 | *LY96* | 1.21 | 0.135886 | *RIPK2* | -1.03 | 0.481575 |
| *CCL5* | -1.09 | 0.498349 | *LYZ* | -1.13 | 0.718865 | *SLC11A1* | 1.31 | **0.032856** |
| *CD14* | -1.00 | 0.899548 | *MAP2K1* | 1.11 | 0.072455 | *SLPI* | 1.05 | 0.509656 |
| *CHUK* | 1.00 | 0.905933 | *MAP2K3* | 1.26 | **0.035239** | *SUGT1* | 1.34 | **0.002965** |
| *CRP* | -1.36 | 0.479025 | *MAP2K4* | -1.1 | **0.031527** | *TICAM1* | 1.21 | 0.113714 |
| *CTSG* | -1.76 | 0.183773 | *MAP3K7* | 1.19 | 0.066373 | *TICAM2* | 1.03 | 0.585415 |
| *CXCL1* | -1.16 | 0.149332 | *MAPK1* | 1.03 | 0.711515 | *TIRAP* | -1.05 | 0.681702 |
| *CXCL2* | -1.10 | 0.376165 | *MAPK14* | -1.01 | 0.885853 | *TLR1* | -1.28 | 0.308421 |
| *DMBT1* | 1.30 | 0.156988 | *MAPK3* | 1.05 | 0.355827 | *TLR2* | -1.18 | **0.046925** |
| *FADD* | 1.20 | 0.163872 | *MAPK8* | 1.04 | 0.321486 | *TLR4* | 1.19 | 0.28294 |
| *HSP90AA1* | 1.07 | 0.477831 | *MEFV* | 1.28 | 0.127412 | *TLR5* | -1.72 | 0.16315 |
| *IFNA1* | -1.22 | 0.775638 | *MPO* | 1.24 | 0.104408 | *TLR6* | 1.01 | 0.950669 |
| *IFNB1* | -1.01 | 0.854786 | *MYD88* | 1.02 | 0.725687 | *TLR9* | 2 | 0.567268 |
| *IKBKB* | -1.05 | 0.776203 | *NAIP* | -1.24 | **0.046051** | *TNF* | -1.14 | 0.46006 |
| *IL12A* | -1.00 | 0.928584 | *NFKB1* | 1.06 | 0.542382 | *TNFRSF1A* | 1.49 | 0.158308 |
| *IL12B* | -1.24 | 0.300942 | *NFKBIA* | -1.02 | 0.773399 | *TOLLIP* | 1.17 | 0.070796 |
| *IL18* | 1.07 | 0.423868 | *NLRC4* | 1.02 | 0.942593 | *TRAF6* | 1.14 | 0.403881 |
| *IL1B* | -1.40 | **0.039549** | *NLRP1* | -1.09 | 0.54494 | *XIAP* | 1.06 | 0.55719 |
| *IL6* | -1.38 | **0.020796** | *NLRP3* | 1.01 | 0.893562 | *ZBP1* | 2.36 | 0.346567 |

**Supplemental Table 1.** Antibacterial response RT-PCR array in primary human urothelial cells transiently transfected with non-targeting control or PTEN siRNA pools and subsequently challenged with UPEC (strain UTI89) for two hours. Shown are the fold regulation comparing transcript expression in PTEN silenced cells relative to controls (*n*=3/group). *P-*values <0.05 are shown in bold. **These data supplement Figure 2A.**
