# Supplementary material for: PTEN modulates urinary tract infection susceptibility and shapes urothelial antibacterial defenses
Source: Life Sci Alliance. 2025 Jul 23;8(10):e202503292. doi: 10.26508/lsa.202503292 (PMC12287727; doi:10.26508/lsa.202503292)
Supplement: Supplementary file 2 [file LSA-2025-03292_TableS2.docx]

| **Reagent** | **Vendor** | **Catalog Number** |
| --- | --- | --- |
| Mice | | |
| Mouse: UPK2 iCre: B6;CBA-Tg(Upk2-icre/ERT2)1Ccc/J | The Jackson Laboratory | JAX: 024768 |
| Mouse: B6.129S4-*Pten^tm1Hwu^*/J | The Jackson Laboratory | JAX: 006440 |
| Cell Lines | | |
| Primary human urothelial cells (HBLAK) | CELLnTEC Advanced Cell Systems | Cat#HBLAK  RRID:CVCL_JQ59 |
| Bacteria | | |
| Uropathogenic *Escherichia coli* UTI89 | Provided by Dr. Scott J. Hultgren | PMID: 2877947 |
| Antibodies | | |
| GAPDH | Cell Signaling Technology | Cat#2118 |
| P-p65 (Ser536) | Cell Signaling Technology | Cat#3033 |
| P-AKT (Ser473) | Cell Signaling Technology | Cat#4060 |
| P-FAK (Y397) | Abcam | Cat#ab81298 |
| PTEN | Cell Signaling Technology | Cat#9188 |
| Ly6G-PerCP Cy5.5 | Biolegend | Cat#127616 |
| CD45-BV780 | Biolegend | Cat#103149 |
| MHC II-BV650 | Biolegend | Cat#107641 |
| Ly6C-eF405 | Invitrogen | Cat#48-5932-82 |
| LiveDead Blue | Invitrogen | Cat#L23105 |
| CD11b-APC | Biolegend | Cat#101212 |
| CX3CR1-PE | Biolegend | Cat#149006 |
| Chemical Reagents | | |
| Absolute Blue QPCR SYBR Mix | Thermo-Fisher | Cat#AB4322B |
| DharmaFECT transfection reagent | Dharmacon | Cat#T-2001-02 |
| DMSO | Fisher Scientific | Cat#D128-1 |
| bpV(HOpic) | Selleckchem | Cat#S8651 |
| Lipofectamine | Thermo-Fisher | Cat#15338100 |
| Tamoxifen | Cayman Chemical | Cat#13258 |
| Commercial Assays | | |
| RNAscope™ 2.5 HD Duplex Assay | Advanced Cell Diagnostics | Cat#322436 |
| *DapB*-C2 prob | Advanced Cell Diagnostics | Cat#320751-C2 |
| FAK [pY397] ELISA | Invitrogen | Cat#KHO0441 |
| Pathscan Phospho-NFkB p65 (Ser536) ELISA | Cell Signaling Technology | Cat#7173 |
| RNAscope® Probe - Mm-Pten-E5 | Advanced Cell Diagnostics | Cat#464331 |
| RNeasy Plus Mini Kit | Qiagen | Cat#74134 |
| Verso cDNA synthesis kit | Thermo-Fisher | Cat#AB1453A |
| RT2 First Strand Kit | Qiagen | Cat#330404 |
| RT2 SYBR Green ROX qPCR Mastermix | Qiagen | Cat# 330523 |
| Human Antibacterial Response Arrays | Qiagen | 330231, PAHS-148Z |
| siRNA Pools | | |
| SMARTpool: ON-TARGETplus *NFKB1* siRNA | Horizon Discovery | L-003520-00-0005 |
| SMARTpool: Non-Targeting siRNA Pool | Horizon Discovery | D-001810-10-05 |
| SMARTpool: ON-TARGETplus *PTEN* siRNA | Horizon Discovery | L-003023-00-0005 |
| SMARTpool: ON-TARGETplus *PTK2* siRNA | Horizon Discovery | L-003164-00-0005 |

**Supplemental Table 2:** Mice and key reagents.
