# Supplementary material for: PTEN modulates urinary tract infection susceptibility and shapes urothelial antibacterial defenses
Source: Life Sci Alliance. 2025 Jul 23;8(10):e202503292. doi: 10.26508/lsa.202503292 (PMC12287727; doi:10.26508/lsa.202503292)
Supplement: Supplementary file 3 [file LSA-2025-03292_TableS3.docx]

| Category | Definition | Score | | Max Score |
| --- | --- | --- | --- | --- |
| **Urothelium** | | | | |
| Number of urothelial cell layers | Maximum number of urothelial cells lining mucosa | 0 | 2-3 cell layers (normal) | 2 |
|  |  | 1 | 4 cell layers |  |
|  |  | 2 | 5+ cell layers |  |
| Ballooning Degeneration | Urothelial cell cytoplasmic swelling and clearing | 0 | Absent | 1 |
|  |  | 1 | Present |  |
| Necrosis/Apoptosis | Dead or dying urothelial cell and sloughing into lumen | 0 | Absent | 1 |
|  |  | 1 | Present |  |
| Mitoses | Mitotic figures observed in urothelial cells | 0 | Absent | 1 |
|  |  | 1 | Present |  |
| Neutrophil Transmigration | Neutrophils present within the urothelium | 0 | Absent | 1 |
|  |  | 1 | Present |  |
| Bacteria | Bacteria noted within lumen and/or attached to urothelium | 0 | Absent | 1 |
|  |  | 1 | Present |  |
| Intracellular Bacterial Communities (IBCs) | Bacteria within urothelium forming discrete cytoplasmic colonies (sum of each quadrant of urinary bladder) | 0 | Absent | 4 |
|  |  | 1 | Present |  |
| **Lamina Propria and Muscularis** | | | | |
| Edema | Increases separation of lamina propria +/- muscularis (sum of each quadrant of urinary bladder) | 0 | No edema present | 8 |
|  |  | 0.5 | Lamina propria edema < width of urothelium (U) + muscularis (M) |  |
|  |  | 1 | Lamina propria edema > width of U + M but < 2x width of U + M |  |
|  |  | 1.5 | Lamina propria edema 2-4x width of U + M |  |
|  |  | 2 | Lamina propria edema > 4x width of U + M |  |
| Inflammation | Neutrophils in lamina propria +/- muscularis (sum of each quadrant of urinary bladder) | 0 | Absent | 4 |
|  |  | 1 | Present |  |
| Hemorrhage | Aggregates of free erythrocytes in lamina propria +/- muscularis (sum of each quadrant of urinary bladder) | 0 | Absent | 4 |
|  |  | 1 | Present |  |
| Lymphoid Nodule Formation | Clusters of lymphocytes forming nodules in the lamina propria | 0 | Absent | 1 |
|  |  | 1 | Present |  |
| **Serosa** | | | | |
| Serosal Mesothelial Hypertrophy | Rounding and enlargement of serosal mesothelium | 0 | Absent | 1 |
|  |  | 1 | Present |  |

**Supplemental Table 3**. Mouse bladder histopathology scoring.
