# Supplementary material for: PTEN modulates urinary tract infection susceptibility and shapes urothelial antibacterial defenses
Source: Life Sci Alliance. 2025 Jul 23;8(10):e202503292. doi: 10.26508/lsa.202503292 (PMC12287727; doi:10.26508/lsa.202503292)
Supplement: Supplementary file 4 [file LSA-2025-03292_TableS4.docx]

| **qRT-PCR** | | |
| --- | --- | --- |
| **Gene** | **5’ Primer** | **3’ Primer** |
| *ACTN1* | CAACGACCCCCAGAAGAAGA | GCCTGGAATGTCACTACCC |
| *CDH1* | GTGCCTGAGAACGAGGCTAA | GTGTATACAGCCTCCCACGC |
| *CLDN4* | CCACTCGGACAACTTCCCAA | ACTTCCGTCCCTCCCCAATA |
| *GAPDH* | TGCACCACCAACTGCTTAGC | GGCATGGACTGTGGTCATGAG |
| *ITGA2* | ATTCTCCCTGCCGGTTGATG | CAGGGTAGCCTACATCGCAG |
| *ITGA3* | TATGGGCGCGGTGTTCTTG | AGGTCCTGCCACCCATCATT |
| *ITGB1* | CCGCGCGGAAAAGATGAAT | CCACAATTTGGCCCTGCTTG |
| *ITGB4* | CTGCAGCCCCATCTCCTAGC | GCCATCCTCTTCCTCCCTCT |
| *NFKB1* | CAGGAAGAGGAGGTTTCGCCA | AGCTGTCAGCGCGTCG |
| *PTEN* | TGTAAAGCTGGAAAGGGACGA | GGAATAGTTACTCCCTTTTTGTCTC |
| *PTK2* | GCAGCTCCACCAAAGAAACC | CTGAAGCTTGACACCCTCGT |
| *PXN* | TCCCTGCCATCACTGTGAAC | CAGGCCCTGGATCTTGAAAT |
| *TJP1* | TTTGGTGATGTGTGGTCCCC | ATCACAGTGTGGTAAGCGCA |
| *UPK1A* | CATTCTTGCTGAACCGTTTGTG | GTGACCGTGACAGAACTCTCATG |
| *UPK1B* | CGCTTGCCTTCAGCTTGTG | GGCCCTGGAAGCAACGA |
| *UPK2* | CAGTGCCTCACCTTCCAACA | TGGTAAAATGGGAGGAAAGTCAA |

**Supplemental Table 4**: List of human primer sequences
