# Supplementary material for: PTEN modulates urinary tract infection susceptibility and shapes urothelial antibacterial defenses
Source: Life Sci Alliance. 2025 Jul 23;8(10):e202503292. doi: 10.26508/lsa.202503292 (PMC12287727; doi:10.26508/lsa.202503292)
Supplement: Supplementary file 5 [file LSA-2025-03292_TableS5.docx]

| **Genotyping** | | |
| --- | --- | --- |
| **Gene** | **5’ Primer** | **3’ Primer** |
| *Pten* flox | CAAGCACTCTGCGAACTGAG | AAGTTTTTGAAGGCAAGATGC |
| UPK2-iCre | CGGGTCAGAAAGAATGGTGT | TCTCTGCCCAGAGTCATCCT |
| *Actb* | ATCGATGCCGGTGCTAAGAA | TCCTATGGGAGAACGGCAGA |
| **qRT-PCR** | | |
| *Cdh1* | ATCCTCGCCCTGCTGATT | CTTTCGAGCCCCCGCTTT |
| *Cldn4* | TTTTGTGGTCACCGACTTTG | TGTAGTCCCATAGACGCCATC |
| *Cldn8* | CTTTCCCTCTACACGCCTTAAT | AAGACCACTGCTTAGCCTTAC |
| *Cxcl1* | GGTGTCCCCAAGTAACGGAG | TTGTCAGAAGCCAGCGTTCA |
| *Cxcl2* | GCTGTCCCTCAACGGAAGAA | CAGGTACGATCCAGGCTTCC |
| *Gapdh* | CTGGAGAAACCTGCCAAGTA | TGTTGCTGTAGCCGTATTCA |
| *Il1* | TGCCACCTTTTGACAGTGATG | ATACTGCCTGCCTGAAGCTC |
| *Il6* | AGTCCTTCCTACCCCAATTTCC | GGTCTTGGTCCTTAGCCACT |
| *Pten* | TGAAGACCATAACCCACCACAGC | CACCAGTCCGTCCCTTTCCA |
| *Tjp1* | GGTTTTGTCTCATCATTTCTTCAG | TGCAGACCCAGCAAAGGT |
| *Tnf* | ATGGCCTCCCTCTCATCAGT | TTTGCTACGACGTGGGCTAC |
| *Upk1b* | GCCATCCTCTGCTGGACTT | AGCACCAAATTCAGAGTCACC |
| *Upk2* | GTTCCCAGCGCAGTATGG | GAGAGGCTTGAGATGTTGAAGTC |
| *Upk3* | AGTTGGGGCTGGAGGTTC | ATCGCGAAGGCTCATCTC |
| **RT-PCR** | | |
| *Pten* ∆Exon5 | ACACCGCCAAATTTAACTGC | GGGTCCTGAATTGGAGGAAT |

**Supplemental Table 5**: List of mouse primer sequences
